# Supplementary material for: The Expression and Function of Notch Involved in Ovarian Development and Fecundity in Basilepta melanopus
Source: Insects. 2024 Apr 19;15(4):292. doi: 10.3390/insects15040292 (PMC11050577; doi:10.3390/insects15040292)
Supplement: Supplementary file 1 [file insects-15-00292-s001.zip › insects-2952828-supplementary.pdf]

Supplementary Materials

The following supporting information can be downloaded at:

Table S1. List of primers used for qPCR and dsNotch synthesis.;

Primer	Sequence（5′-3′）
Notch-01F	GTGTCGGCAAATGGAGAA
Notch-01R	GACTGGTGTCGCAAATCG
EGFP-S2F	TGCCCATCCTGGTCGAGCT
EGFP-S2R	TGCTTGTCGGCCATGATAT
T7-01 F	GGATCCTAATACGACTCACTATAGGGTGTCGGCAAATGG AGAA
T7-01 R	GGATCCTAATACGACTCACTATAGGGACTGGTGTCGCAA ATCG
T7-EGFP-S2F	GGATCCTAATACGACTCACTATAGGTGCCCATCCTGGTC GAGCT
T7-EGFP-S2R	GGATCCTAATACGACTCACTATAGGTGCTTGTCGGCCAT GATAT
Notch-Q1F	AAACCTACCAAGTCCCTA
Notch-Q1R	CCATCTAAAGTAAACCCTAA
β-actin-Q1F	CGCTTCTGGTCGTACAACTG
β-actin-Q1F	TGGTGAATGAGTAGCCACGT

Table S2: Information for specimens used in the phylogenetic analysis.

Order	Species	GenBank ID
Coleoptera	*Diabrotica virgifera virgifera*	XP_028149569.1
	Anoplophora glabripennis	XP_018574509.1
	Leptinotarsa decemlineata	XP_023013802.1
	Psylliodes chrysocephala	CAH1109257.1
	Tenebrio molitor	CAH1379345.1
	Tribolium castaneum	XP_008200304.1
Diptera	*Drosophila melanogaster*	NP_001245510.1
	Aedes aegypti	EAT37841.1
	Toxorhynchites rutilus septentrionalis	XP_055615510
	Malaya genurostris	XP_058444774.1
	Bactrocera tryoni	XP_039958472.1
Orthoptera	*Schistocerca gregaria*	XP_049844801.1
	Locusta migratoria migratoria	AXY96273.1
	Gryllus bimaculatus	GLG99742.1
	Schistocerca cancellata	XP_049784050.1
Lepidoptera	*Helicoverpa zea*	XP_047028405.1
	Spodoptera frugiperda	XP_050553762.1
	Bombyx mori	NP_001157370.1
	Pararge aegeria	XP_039765579.1
	Arctia plantaginis	CAB3259242.1
	Chilo suppressalis	CAH0691090.1
	Tuta absoluta	KAJ2946674.1
Hymenoptera	*Athalia rosae*	XP_012252830.2
	Colletes gigas	XP_043254354.1
	Polistes dominula	XP_015172000.1
	Vespa velutina	XP_047357738.1
	Osmia bicornis	XP_029033765.1
	Megachile rotundata	XP_012145055.1
	Cataglyphis hispanica	XP_050453731.1
